# A Novel *Phytophthora sojae* Resistance *Rps12 *Gene Mapped to a Genomic Region That Contains Several *Rps* Genes

**DOI:** 10.1371/journal.pone.0169950

**Published:** 2017-01-12

**Authors:** Dipak K. Sahoo, Nilwala S. Abeysekara, Silvia R. Cianzio, Alison E. Robertson, Madan K. Bhattacharyya

**Affiliations:** 1 Department of Agronomy, Iowa State University, Ames, IA, United States of America; 2 Department Plant Pathology and Microbiology, Iowa State University, Ames, IA, United States of America; Nanjing Agricultural University, CHINA

## Abstract

*Phytophthora sojae* Kaufmann and Gerdemann, which causes *Phytophthora* root rot, is a widespread pathogen that limits soybean production worldwide. Development of *Phytophthora* resistant cultivars carrying *Phytophthora* resistance *Rps* genes is a cost-effective approach in controlling this disease. For this mapping study of a novel *Rps* gene, 290 recombinant inbred lines (RILs) (F_7_ families) were developed by crossing the *P*. *sojae* resistant cultivar PI399036 with the *P*. *sojae* susceptible AR2 line, and were phenotyped for responses to a mixture of three *P*. *sojae* isolates that overcome most of the known *Rps* genes. Of these 290 RILs, 130 were homozygous resistant, 12 heterzygous and segregating for *Phytophthora* resistance, and 148 were recessive homozygous and susceptible. From this population, 59 RILs homozygous for *Phytophthora sojae* resistance and 61 susceptible to a mixture of *P*. *sojae* isolates R17 and Val12-11 or P7074 that overcome resistance encoded by known *Rps* genes mapped to Chromosome 18 were selected for mapping novel *Rps* gene. A single gene accounted for the 1:1 segregation of resistance and susceptibility among the RILs. The gene encoding the *Phytophthora* resistance mapped to a 5.8 cM interval between the SSR markers BARCSOYSSR_18_1840 and Sat_064 located in the lower arm of Chromosome 18. The gene is mapped 2.2 cM proximal to the *NBSRps4/6*-like sequence that was reported to co-segregate with the *Phytophthora* resistance genes *Rps4* and *Rps6*. The gene is mapped to a highly recombinogenic, gene-rich genomic region carrying several nucleotide binding site-leucine rich repeat (NBS-LRR)-like genes. We named this novel gene as *Rps12*, which is expected to be an invaluable resource in breeding soybeans for *Phytophthora* resistance.

## Introduction

*Phytophthora* root and stem rot (PRR), caused by *Phytophthora sojae* Kaufmann and Gerdemann, is one of the most devastating diseases in soybean [*Glycine max* (L.) Merr.] [1]. The disease was first reported in Indiana in 1948, in Ohio in 1951, and subsequently spread to all soybean-growing regions of the United States (US) [2]. It is most prevalent in the North Central region where the environmental conditions favor disease development [3]. *P*. *sojae* has also been reported in other soybean-growing countries, including Argentina, Brazil, China, Japan, Indonesia, Australia, Canada, and Europe [4–9]. The estimated annual yield suppression from the disease has been valued at $200 million in the North Central United States, and approximately $1–2 billion worldwide [10–11].

Though the soil-borne oomycete *P*. *sojae* primarily attacks soybean seedlings prior to emergence [1], disease can occur at any stage of plant development and throughout the growing season. Disease symptoms include brown stem lesions that develop in the roots and gradually progress to the stems, followed by wilting, chlorosis, and plant death [12]. In addition, plants infected with *P*. *sojae* may become more vulnerable to infection by other soil-borne pathogens. *P*. *sojae* can survive as mycelia or as oospores in soil or soybean plant debris for many years without a host. Under saturated soil conditions, especially during warm and wet weather, oospores germinate and produce sporangia containing hundreds of small, mobile spores called zoospores, which swim through the water-filled soil pores and infect soybean roots [1, 8, 13]. Epidemics of PRR usually occur in poorly drained fields because flooded fields or saturated soil favor sporulation and dissemination of zoospores [1].

Soybean cultivars and germplasm accessions differ in their responses to isolates of *P*. *sojae* [2]. The use of resistant soybean cultivars is the most economical and effective method of controlling this pathogen. Two distinct types of host resistance to *P*. *sojae* have been described: (i) race-specific resistance conditioned by single dominant genes (*Rps*); and (ii) broad-spectrum partial non-race-specific resistance conferred by several minor genes [14–15].

When novel *Rps* genes are introduced through the release of new cultivars *P*. *sojae* isolates evolve to overcome the introduced resistance genes [16–17]. Over 200 known pathotypes of this pathogen have been reported [18–19], presumably due to selection pressure on the *P*. *sojae* population for new pathotypes that can overcome *Rps* genes [20]. The rapid evolution of new *P*. *sojae* virulent pathotypes limits the effectiveness of an *Rps* gene to 8–15 years [1]. Consequently, there is a constant need for novel *Rps* genes that can effectively manage the disease.

The first *Rps* gene was identified in the 1950s [21]. To date, 27 *Rps* genes have been identified and mapped to eight chromosomes (S1 Table). The *Rps* genes encode receptors that presumably recognize *P*. *sojae* effectors and induce effector-triggered immunity [22]. The *Rps* genes mapped to Chromosome 3 include *Rps1*, *Rps7*, *Rps9*, *RpsYu25*, *RpsYD29*, *RpsYD25*, *RpsUN1* and *Rps1*? [14, 23–31]. The *Rps1* locus is complex and contains at least five functional alleles, *Rps1a*, *1b*, *1c* and *1d* and *1k* [28, 32–33]. High resolution genetic and physical maps were constructed for the *Rps1-*k region and two functional nucleotide binding site-leucine rich repeat (NBS-LRR) containing *Rps* genes, *Rps1-*k-1 and *Rps1-*k-2, were cloned from the *Rps1-*k locus [29, 34–37]. Recent studies have revealed that additional alleles may be present in the *Rps1* locus. For example, *Rps1*? gene in Waseshiroge, *RpsYu25* and *RpsYD25* in the Chinese cultivar ‘Yudou 25’, and *Rps9* in the Chinese cultivar ‘Ludou 4’ have been considered to be either allelic to *Rps1* or *Rps1-*linked genes [14, 38–39]. The *Rps2* gene and *RpsUN2* have been mapped to Chromosome 16 [27, 40–41]. Three *Rps3* alleles, *Rps3a*, *Rps3b* and *Rps3c*, *Rps8* and *RpsSN10* have been mapped to Chromosome 13 [27, 42–48]. Although earlier studies suggested no linkage between *Rps4* and *Rps6* [49], *Rps4*, *Rps5*, *Rps6* and *RpsJS* are tightly linked genes that are located on the lower arm of Chromosome 18 [27, 50–54]. In fact, *Rps4* and *Rps6* could be allelic [50]. *Rps10* has been mapped to Chromosome 17 [55], *RpsYB30* and *RpsZS18* [56–57] to Chromosome 19 and Chromosome 2, respectively, and *Rps11* to Chromosome 7 [58].

An earlier study [59] suggested that PI399036 contains multiple *Rps* genes including at least one novel *Rps* gene. Our recent mapping study of quantitative trait loci underlying partial resistance to *P*. *sojae* [60] using a mixture of three *P*. *sojae* isolates suggested the presence of a putative novel *Rps* gene on the lower arm of Chromosome 18. The present study was undertaken to map this potential novel *Rps* gene. We observed that a single dominant *Phytophthora* resistance gene, named *Rps12*, is tightly linked to the proximal side of the *Rps4/6* locus in a 5.4 cM region between the SSR marker BARCSOYSSR_18_1840 and the NBSRps4/6-130/533 sequence.

## Materials and Methods

### Plant genetic material

The AX20925 recombinant inbred line (RIL) population was developed by crossing PI399036 (USDA-ARS National Soybean Germplasm Collection) with the germplasm line AR2, released by Iowa State University (S.R. Cianzio, D.R. Charlson, G. Gebhart, N. Rivera, P. Lundeen, and R. Shoemaker, unpublished). The cross was made at the Iowa State University research site at the University of Puerto Rico’s Isabela Substation (ISU-PR) [60].

The individual F_2_ plants were advanced to the F_6_ generation by applying single-seed descent breeding method. One hundred seeds of each individual F_6_ plant were planted and harvested in bulk to obtain F_7_ seeds [recombinant inbred line (RILs)] used in this study [60]. In this study, 290 F_7_ families [recombinant inbred lines (RILs)] were phenotyped for responses to a mixture of three *P*. *sojae* isolates [60] that overcome most of the known *Rps* genes. Of these 290 RILs, 130 were homozygous resistant, 12 heterzygous for *Phytophthora* resistance and 148 were recessive homozygous and susceptible. In this molecular mapping study, 120 RILs of the 290 RILs were investigated. Eleven plants each from selected 120 RILs were scored again for responses to the *P*. *sojae* isolates in each of the three independent experiments. Among these 120 RILs, 59 were homozygous resistant and 61 were susceptible to the pathogen.

### *Phytophthora sojae* isolates

*Phytophthora sojae* R17 (*vir 1b*, *1d*, *3a*, *3b*, *3c*, *5*, *6*), Val 12–11 (*vir 1a*, *1b*, *1c*, *1d*, *1k*, *2*, *4*, *7*), and P7074 *(vir 1b*, *1d*, *2*, *3a*, *3b*, *3c*, *4*, *5*, *6*, *7*, *8)* isolates were used in this study (Table 1). *Phytophthora sojae* isolate R17 was obtained from Dr. Anne Dorrance (Ohio State University, OH), Val 12–11 from Dr. Martin Chilvers (Michigan State University, MI) and strain P7074 from Dr. Alison E. Robertson (Iowa State University). All isolates were grown on half strength V8 agar plates amended with neomycin sulfate and chloramphenicol antibiotics for 5–7 days under room temperature in the dark as described by Dorrance et al. [12].

**Table 1:** Reactions of soybean differentials carrying *Rps1a*, *1b*, *1c*, *1d*, *1k*, *2*, *3a*, *3b*, *3c*, *4*, *5*, *6*, *7*, and *8* genes to *Phytophthora sojae* isolates.

Differential Line	*Rps gene*	R17	Val12-11	R17 & Val12-11	P7074
**L88-8470**	***1a***	**0**	**100**	**80–100**	**0–5**
**L77-1863**	***1b***	**83–100**	**100**	**86–100**	**96–100**
**Williams 79**	***1c***	**0–13**	**100**	**86–100**	**0**
**L93-3312**	***1d***	**100**	**100**	**88–100**	**100**
**Williams 82**	***1k***	**0**	**100**	**80–100**	**0–10**
**L82-1449**	***2***	**33**	**90–100**	**71–100**	**80–100**
**L83-570**	***3a***	**100**	**0**	**100**	**93–100**
**L91-8347**	***3b***	**100**	**0**	**80–100**	**96–100**
**L92-7857**	***3c***	**100**	**17**	**100**	**88–100**
**L85-2352**	***4***	**17**	**100**	**100**	**90–100**
**L85-3059**	***5***	**100**	**11**	**88–100**	**95–100**
**L89-1581**	***6***	**100**	**0**	**86–100**	**85–100**
**L93-3258**	***7***	**50–67**	**100**	**100**	**93–100**
**PI 399073**	***8***	**33**	**13**	**31–67**	**77–100**
**Sloan**	*** ***	**100**	**100**	**100**	**100**

R17, *P*. *sojae* R17 isolate; Val12-11, *P*. *sojae* Val12-11 isolate; R17+Val12-11, a mixture of *P*. *sojae* R17 and Val12-11 isolates; P7074, *P*. *sojae* strain P7074 alone, Data are in % dead seedlings.

### Evaluation of genetic materials for *phytophthora* resistance

The 120 RILs, the parents PI399036 and AR2 along with 14 differential lines and the susceptible cultivar ‘Sloan’ [12, 61] with no known *Rps* genes were planted in vermiculite filled 237 mL Styrofoam cups (11 seeds per cup) and watered once a day. The differential lines include lines that carry *Rps1a*, *Rps1b*, *Rps1c*, *Rps1d*, *Rps1k*, *Rps2*, *Rps3a*, *Rps3b*, *Rps3c*, *Rps4*, *Rps5*, *Rps6*, *Rps7*, and *Rps8* genes [19, 62]. Seedlings were grown in the greenhouse for a week. Hypocotyls of seven-day old seedlings were inoculated using the wounded-hypocotyl inoculation technique [18–20, 59–63]. An approximately 1 cm long slit was made with the needle tip in each hypocotyl, 1 cm below the cotyledonary node, and 0.2 to 0.4 mL of the culture slurry was placed into the slit using the syringe. Plants were kept in a dew chamber at 25°C for 24 h in the dark after inoculations and then moved to a growth chamber at 25°C with a 12 h photoperiod with light intensity 580 ± 75 μ mol PAR m^-2^ s^-1^. The experiment was repeated two more times. Plants were rated seven days after inoculation as either R (resistant, <30% seedling death) or S (susceptible, ≥70% seedling death).

**Fig 1:**
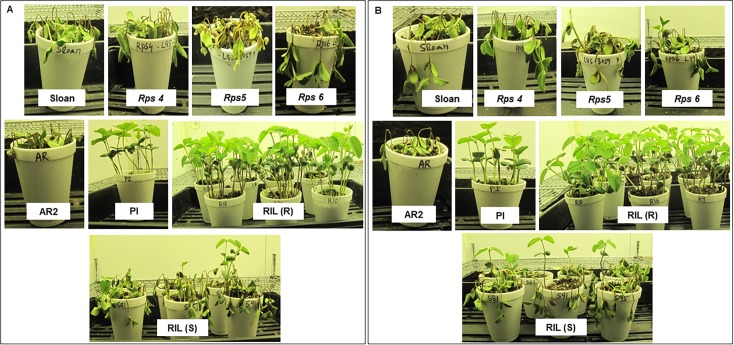
**Reactions of soybean differentials carrying *Rps4*, *Rps5*, and *Rps6* genes and RILs with or without the novel *Rps* gene to (A) a mixture of *P*. *sojae* of R17 and Val12-11 isolates, and (B) the *P*. *sojae* P7074 isolate.** The presence of a dying or expanded lesion indicates a susceptible response or compatible interaction. Resistance response is expressed as hypersensitive cell death at the inoculation sites and healthy nature of infected plants. AR2, susceptible parent AR2; PI, resistant parent PI399036 containing the novel *Rps* gene; RIL (R), randomly selected recombinant inbred lines resistant to *P*. *sojae* isolates; RIL (S), randomly selected recombinant inbred lines susceptible to *P*. *sojae* isolates; Sloan, the susceptible cultivar Sloan with no known *Rps* genes used as the susceptible control.

Inocula were prepared using a modified version of the protocol described by Dorrance et al. [12]. Isolates were grown on soft V8 juice agar (12 g agar/liter) at 22°C under dark conditions until the mycelia covered the entire plate. The colonized agar was cut in strips, placed in a 10-mL syringe and forced out through the syringe to prepare inoculum pulp. The macerated culture was placed in a syringe for a second time and a #18 needle was used to further macerate the culture. Macerated R17 and Val 12–11 cultures were mixed in a 1:1 ratio to prepare the mixed inoculum [63], which is virulent to soybean cultivars carrying *Rps* genes mapped to any of the *Rps1* to *7* loci (Fig 1, Table 1). *P*. *sojae* strain P7074 [22, 64–65] was also used as a separate source of inoculum as it is virulent to soybean lines carrying *Rps4*, *5* and *6* (Fig 1).

### DNA preparation and bulked segregant analysis (BSA)

Prior to inoculation, one unifoliate leaf from each of 11 random plants per RIL was harvested, bulked and frozen in liquid nitrogen, and stored at -80°C. The genomic DNA was extracted from the bulked leaf samples using the CTAB (cetyl trimethyl-ammonium bromide) method [66]. To identify microsatellite and molecular markers, we conducted bulked segregant analysis (BSA) [67] using pooled DNA samples of 10 homozygous resistant (Resistant Bulk) or 10 susceptible (Susceptible Bulk) RILs. One μg DNA from each selected RIL was used for pooling. Each DNA bulk was diluted to a final concentration of 50 ng DNA/μL.

### Molecular marker analyses

Microsatellite (simple sequence repeats, SSR) and molecular markers were used to construct a linkage map of the genomic region carrying the putative novel *Rps* gene locus. Molecular markers based on previously reported *NBSRps4/6* sequence [50] were developed for mapping the novel *Rps* gene (S2 Table). SSR primers were synthesized using the sequence data available at SoyBase (http://soybase.org/) (S2 Table). Primer sequences for SSR markers linked to *RpsJS* were obtained from a published report [54] (S2 Table). For SSR analysis, 50 ng DNA extracted from leaf samples of each resistant or susceptible RIL was used as the template in a 25 μL reaction containing 1X PCR reaction buffer (10 mM Tris–HCl, 50 mM KCl, pH 8.3), 2.0 mM MgCl_2_; 0.25 μM of each primer, 200 μM of each dNTP, and 1 U *Taq* DNA polymerase. The polymerase chain reaction (PCR) conditions were as follows: 2 min at 94°C; 35 cycles of 30 s at 94°C, 30 s at primer-specific annealing temperature (S2 Table), 1 min extension at 72°C; followed by 10 min at 72°C. The amplification products were separated on a 4% NuSieve^TM^ 3:1 agarose (Lonza, USA) gel, stained with EtBr and then visualized under UV light using FOTO/Analyst Express Systems (FOTODYNE Incorporated, USA). Thirty-four SSR markers covering the novel *Rps* gene region on Chromosome 18 (S2 Table) were evaluated for possible polymorphisms between the AR2 (susceptible), and PI399036 (resistant) parents, and resistant and susceptible bulks of BSA.

### Linkage map construction and statistical analysis

The Chi square (**χ**2) analysis was performed to check the phenotypic data for goodness-of-fit to a Mendelian segregation ratio using Graphpad (http://www.graphpad.com/quickcalcs). To determine genetic distances, Mapmaker version 3.0 [68] and the Kosambi mapping function [69] were used. Marker order was determined using the log-likelihood (LOD) method with threshold 3.0. The linkage map of molecular markers and the *Rps12* locus was constructed using MapChart 2.3 [70].

## Results

### Identification of a putative novel *Rps* gene

PI399036 has been suggested to carry multiple *Rps* genes including known and unknown *Rps* genes [59, 60, 71]. Our previous study of two independent segregating populations suggested that there is a major *Phytophthora* resistance gene in the *Rps4*/6 region of this accession [60]. Here we determine the inheritance of the putative novel gene by evaluating F_2_ and RILs for segregation of *Phytophthora* resistance against an inoculum mixture of Val 12–11 and R17 isolates, which together are virulent on soybean lines carrying all *Phytophthora* resistance genes mapped to the *Rps1* to *7* loci. We also used the P7074 isolate in screening the RILs because this isolate can overcome the resistance encoded by *Rps4*, *5*, and *6* mapped tightly to the *Rps4*/6 region (Fig 1; Table 1).

Phenotypic evaluation of the 25 F_2_ plants obtained from the cross between PI399036 x AR2 following inoculation with the mixture of the Val 12–11 and R17 isolates resulted in 19 resistant (R) and six susceptible (S) plants. The F_2_ segregation ratio fits the expected 3:1 (R:S) ratio for a single dominant gene for resistance (χ^2^_df = 1_ = 0.013, *p* = 0.908). The screening of the 290 RILs of the AX20925 population with the mixture of the *P*. *sojae* isolates, PT2004 C2.S1 (vir 1a, 1b, 1c, 1d, 1k, 2, 3c, 4, 6,7), 1005–2.9 (vir 1a, 1b, 1c, 1k, 3b, 7), and R7-2a (vir 1d, 2, 3a, 5, 6, 7) [60] resulted in a 130:12:148::R:H(heterozygous):S segregation ratio, which fits the expected 140.5:9:140.5 (R:S) ratio for a single gene segregation among the homozygous RILs (χ^2^_df = 2_ = 2.125, *p* = 0.346).

### Putative mapping of the novel *Rps* gene by BSA

**Fig 2:**
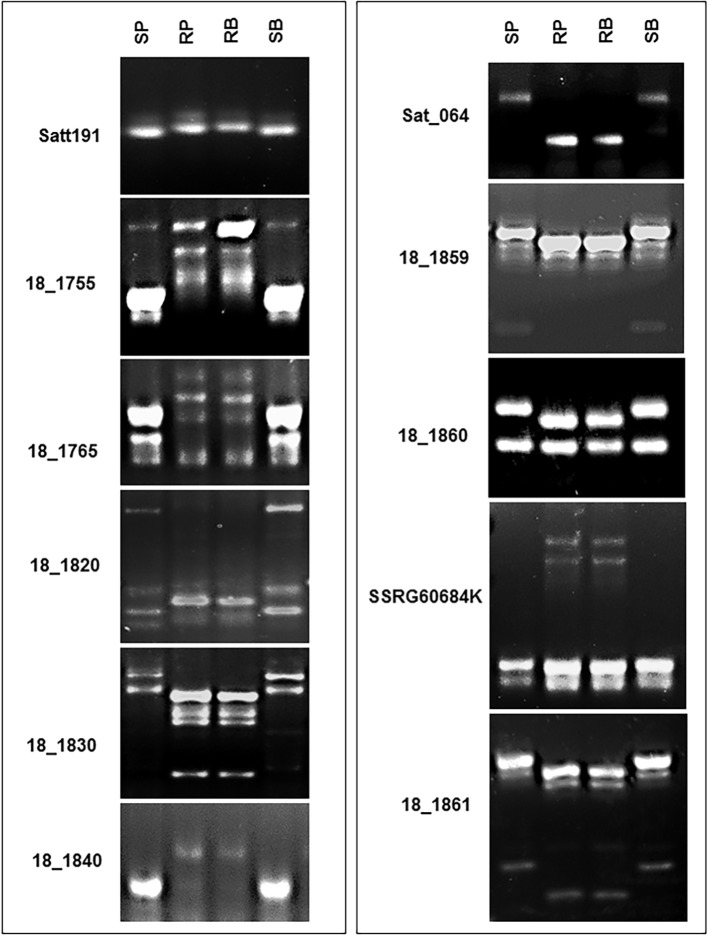
Eleven polymorphic SSR markers linked to *Rps12*. SP, susceptible parent AR2; RP, resistant parent PI399036; RB, bulk of 10 resistant homozygous RILs; SB, bulk of 10 susceptible RILs. Satt191, BARCSOYSSR_18_1750; 18_1755, BARCSOYSSR_18_1755; 18_1765, BARCSOYSSR_18_1765; 18_1820, BARCSOYSSR_18_1820; 18_1830, BARCSOYSSR_18_1830; 18_1840, BARCSOYSSR_18_1840; Sat_064, BARCSOYSSR_18_1858; 18_1859, BARCSOYSSR_18_1859; 18_1860, BARCSOYSSR_18_1860; SSRG60684K, SSRG60684K marker; 18_1861, BARCSOYSSR_18_1861.

To putatively map the novel *Rps* gene, we evaluated 34 SSR markers from the *Rps4*/6 region for polymorphisms among the parents of the population, PI399036 and AR2 (S2 Table). The selected SSR markers encompass the genomic region that includes the *RpsJS*, *Rps4*, and *Rps6* genes [27, 56–57]. Of the 34 SSR markers evaluated, 14 were polymorphic between PI399036 and AR2. These SSR markers were then used to putatively determine map location of the *Rps* gene by conducting BSA [67]. The BSA analysis revealed that the novel *Rps* gene was located in the *Rps4*/6 region. Of the 14 SSR markers, 11 showing close association to the novel *Rps* gene were further considered for mapping the 120 RILs (Fig 2).

**Fig 3:**
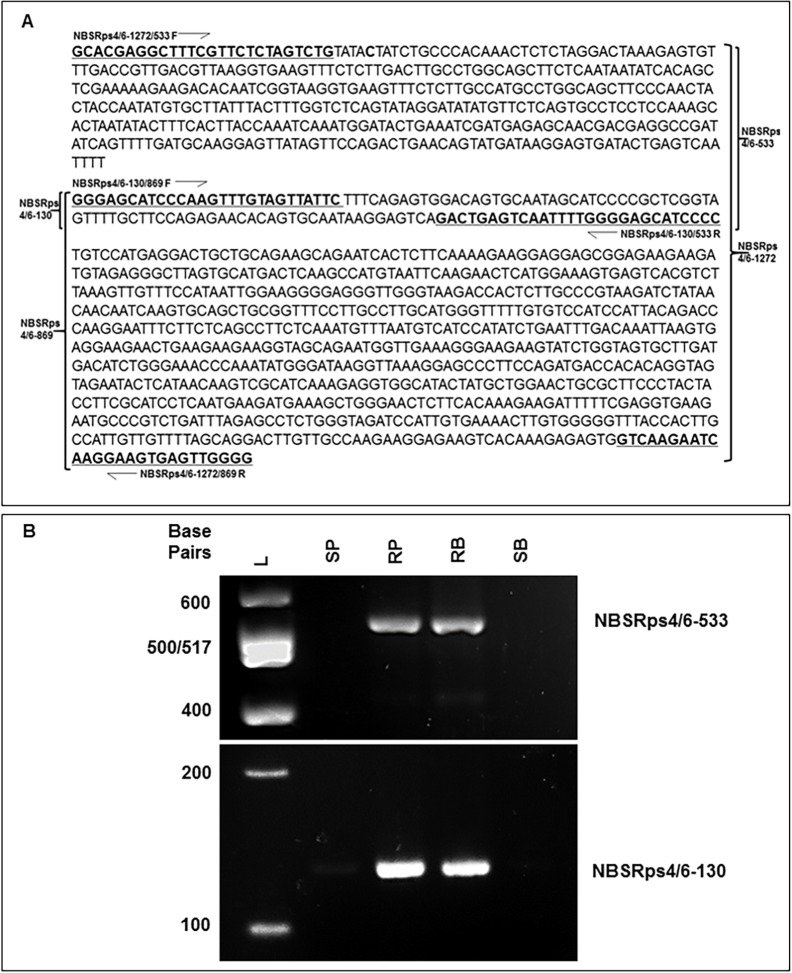
Analysis of *NBSRps4/6*-specific molecular markers linked to a novel *Phytophthora* resistance gene. (A) The *NBSRps4/6* specific sequence (GenBank accession no. AY258630 [50]) used for developing molecular markers. Primer sequences used for PCR are underlined and marked with half arrows. The PCR primers for amplified targets, NBSRps4/6-1272, NBSRps4/6-869, NBSRps4/6-533 and NBSRps4/6-130, are shown along the primers (S2 Table). (B) The *NBSRps4/6* specific molecular markers linked to the novel *Rps* gene. L, 100 bp DNA Ladder (New England Biolabs, USA); SP, susceptible parent AR2; RP, resistant parent PI399036; RB, bulk of 10 resistant homozygous RILs; SB, bulk of 10 susceptible RILs. NBSRps4/6-533, *NBSRps4/6* specific NBSRps4/6-533 marker; NBSRps4/6-130, *NBSRps4/6* specific NBSRps4/6-130 marker.

In addition to the 14 polymorphic SSR markers, we determined if the *NBSRps4/6* sequence previously reported to be the candidate for the *Rps4* gene is polymorphic between the two parents, PI399036 and AR2 [50]. We designed *NBSRps4/6* sequence-specific primers and amplified two PCR products of 130 and 533 bp in length, from both PI399036 and the resistant bulked DNA sample, but not from either AR2 or the susceptible bulked DNA sample (Fig 3). BSA analysis suggested that the amplified *NBSRps4/6-*like sequences co-segregate with the genomic region containing a putative novel *Rps* gene (Fig 3). The 130 bp and 533 bp PCR fragments showed 93% and 99% nucleic acid sequence identity, respectively, to the *NBSRps4/6* sequence reported earlier [50]. The 130 and 533 bp *NBSRps4/6*-type fragments were named as NBSRps4/6-130 and NBSRps4/6-533, respectively.

### Genetic mapping of the novel *Rps* gene

**Fig 4:**
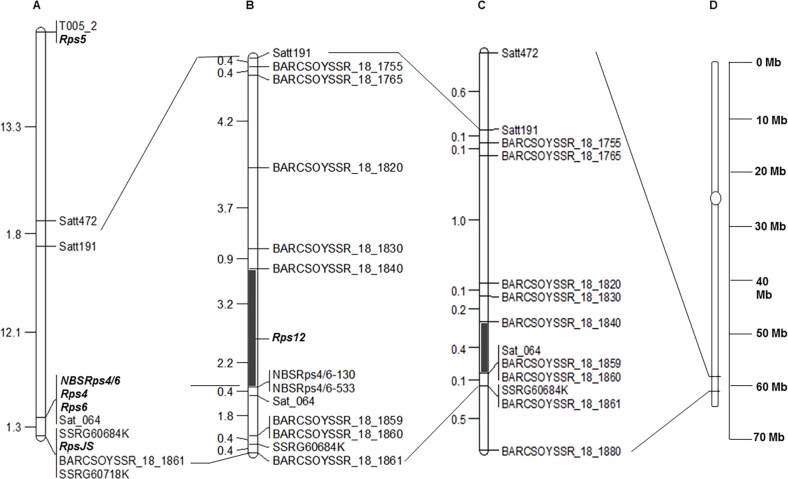
Genetic and physical map of the *Rps12* region. (A) Molecular genetic map of the *Rps* loci of the lower arm of Chromosome 18 (S1D Fig). **(**B) Genetic map of the *Rps12* region. SSR markers are shown on the right side of the map and corresponding genetic distances between two adjacent loci are shown on the left side of the map in centi-Morgan (cM). The *Rps12* region is shown with a solid line. (C) Physical map of SSR markers on Chromosome 18 according to the soybean reference genome sequence (*Glycine max* v1.1: http://soybase.org). The corresponding physical distances between two adjacent loci are shown on the left side of the map in mega base pairs (Mb). The *Rps12* physical region defined by two molecular markers (BARCSOYSSR_18_1820 and Sat_064) is shown by a solid dark line. (D) Physical location of the *Rps12* region on Chromosome 18 (*Glycine max* v1.1: http://soybase.org). The two long bars indicate two arms of Chromosome 18, the circle indicates the approximate position of the centromeric region, and the marked portion indicates the region containing *Rps12*.

Two dominant markers, NBSRps4/6-130 and NBSRps4/6-533, and 11 co-dominant SSR markers (Figs 2 and 3 and S2 Table) from a genomic region of ~3 Mb containing the novel *Rps* gene were used to construct a linkage map. We genotyped all 120 RILs (59 R and 61 S) for the 13 molecular markers (S3 Table). With the genotypic and phenotypic data of the mapping population, a genetic map consisting of the 11 SSR markers, the two dominant markers, NBSRps4/6-130 and NBSRps4/6-533, and the novel *Rps* gene locus was constructed. The new gene was mapped between the SSR markers, BARCSOYSSR_18_1840 and Sat_064 (BARCSOYSSR_18_1858) (Fig 4). Both the NBSRps4/6-130 and NBSRps4/6-533 markers were mapped 2.2 cM distal to the novel *Rps* locus, suggesting that the new *Rps* gene is unlikely to be allelic to *Rps4*. Based on the map positions of the molecular markers linked to previously reported *Rps* genes, it appears that *Rps12* is mapped to a new locus which is distinct from the previously mapped *Rps* loci of the lower arm of Chromosome 18 (Fig 4A and 4B; S1 Fig).

The *RpsJS* gene has also been mapped to the *Rps4*/6 region between the molecular markers, BARCSOYSSR_18_1859 and SSRG60752K [54] (S1 Fig). Both of these markers mapped distal to Sat_064, which co-segregated with the *Rps4/6* locus carrying *Rps4* and *Rps6* genes (S1 Fig). Our mapping data suggest that the novel *Rps* gene is located in the genomic region proximal to the *Rps4*, *6* and *JS* genes and distal to *Rps5*. We conclude that the gene is novel and named the *Phytophthora* resistance gene as *Rps12* (Fig 4).

## Discussion

It has been suggested that PI399036 contains multiple *Rps* genes including known and unknown *Rps* genes [59, 60, 71]. Several major and minor QTL for partial resistance to *P*. *sojae* have also been identified from this accession [60]. Our previous study indicated the presence of a novel *Rps* gene in the *Rps4*/6 region. Responses of the segregating RILs and parents to a *P*. *sojae* isolate and a mixture of two isolates established that the gene is distinct from *Rps4*, *5*, and *6*. In addition to these three *Rps* genes, *RpsJS* was mapped to the lower arm of Chromosome 18 [54]. To determine if the putative novel gene is distinct from *Rps4*, *5*, *6* and *JS*, we investigated the molecular markers that were shown to be linked to these *Phytophthora* resistance genes. The *Rps4* and *Rps6* genes were shown to co-segregate and *Rps4* was tightly linked to Sat_064 [50]. *Rps5* was shown to co-segregate with the RFLP marker T005_2, which is proximal to both the Satt191 and Satt472 SSR markers (Fig 4 and S1 Fig) [53,72]. Therefore, we conclude that the novel *Phytophtora* resistance gene identified in this investigation mapped to the novel locus, *Rps12*.

*Rps12* is located in between two SSR markers, BARCSOYSSR_18_1840 and Sat_064, which span a region of 372 kb DNA. The genetic distance between these two loci is 5.8 cM (Fig 4). These results suggest that the *Rps12* region is highly recombinogenic, with only 64 kb DNA/cM. Thus, introgression of the gene using the BARCSOYSSR_18_1840 and Sat_064 to elite soybean lines would require molecular analyses of a relatively small segregating population (Fig 4). The *Rps12* region contains 45 predicted genes, with on the average one gene in every 8 kb DNA. This means that the highly recombinogenic *Rps12* region is gene-rich (S4 Table). It will therefore be feasible to map the candidate *Rps12* genes to a small genetic interval through use of molecular markers and a large recombinant inbred line population.

Considering the fact that most identified disease resistance genes encode nucleotide binding site-leucine rich repeat (NBS_LRR) containing proteins, we investigated if there are any NBS-LRR-type genes in the *Rps12* region [73]. We identified four clusters of eight NBS-LRR-type genes from this genomic region of the cultivar Williams 82, which has been sequenced (S4 Table) [74]. We observed that although *NBSRps4*/6 is closer to *Rps12* as compared to Sat_064 in the genetic map (Fig 4B), in the Williams 82 genome its physical distance to *Rps12* is larger than the distance between *Rps12* and Sat_064. This could be due to a micro inversion in the Sat_064 region. Alternatively, this could be just an artifact resulting from misassembling of sequences in the Sat_064 region.

The highly recombinogenic nature of the *Rps12* region suggests that positional cloning of the gene could be facilitated through high density mapping of the *Rps12* region using a large segregating population. It is expected that a few of the homozygous RILs for *Rps12* contain QTL conditioned by minor genes for partial *Phytophthora* resistance reported earlier [60] and could be an invaluable germplasm for breeding soybeans.

**Fig 5:**
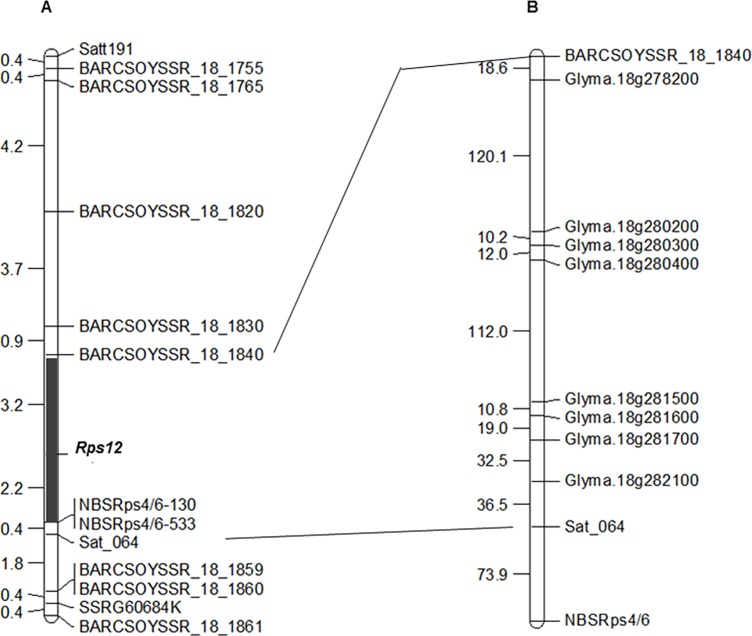
Physical map of NBS-LRR like genes present on the *Rps12* region. (A) Genetic map of the *Rps12* region (Fig 4B). (B) Physical map of eight NBS-LRR-like genes identified from the *Rps12* region of the Williams 82 genome.

In this study, we have demonstrated that the previously identified *Rps4/6* locus is 2.2 cM distal to the *Rps12* locus. To date, *Rps1*-k has been cloned and a strong candidate gene for *Rps4* has been identified. Both encode NBS-LRR genes [29, 50]. Several *Rps* loci have been shown to harbor NBS-LRR sequences, although their functional relevance to *Rps* genes is yet to be established [75–76]. Our data suggest that *Rps12* could be an NBS-LRR-type sequence (Fig 5).

We have evaluated RILs carrying *Rps12* against only three important *P*. *sojae* isolates, which can overcome resistance encoded by most known *Rps* genes (Table 1). The study of RILs for their responses to three isolates indicate that the *Rps12* gene is expected to have agronomical importance in conferring resistance to most *P*. *sojae* isolates that can defeat the *Phytophthora* resistance encoded by currently available *Rps* genes.

## Supporting Information

S1 Fig Genetic map of *Rps* genes on Chromosome 18. (A) The genetic map of the *Rps4/6* region from the study by Sandhu et al. (2004) [50]. (B) The genetic map of the *Rps4* and *Rps5* region from Diers et al. (1992) [53]. (C) The genetic linkage map of the *RpsJS* region from the study of Sun et al. (2014) [54]. (D) The composite genetic map of the *Rps* loci located in the lower arm of Chromosome 18. The map was developed from three maps shown in A, B and C, and the co-segregation of *Rps4* and *Rps6* was from the study of Sandhu et al. (2004) [50].
(TIF)

S1 Table Twenty-seven *Rps* genes that confer resistance to *Phythopthora sojae* in soybean. (DOCX)

S2 Table Primers for microsatellite and NBSRps4/6-sequence-specific markers. (DOC)

S3 Table ^a^Phenotypes and ^b^genotypes of 120 AX20925 RILs. (DOC)

S4 Table GO annotation of the predicted genes of the *Rps12* region. (XLS)
