# Supplementary figures and images for: A Novel Phytophthora sojae Resistance Rps12 Gene Mapped to a Genomic Region That Contains Several Rps Genes
Source: PLoS One. 2017 Jan 12;12(1):e0169950. doi: 10.1371/journal.pone.0169950 (PMC5233422; doi:10.1371/journal.pone.0169950)

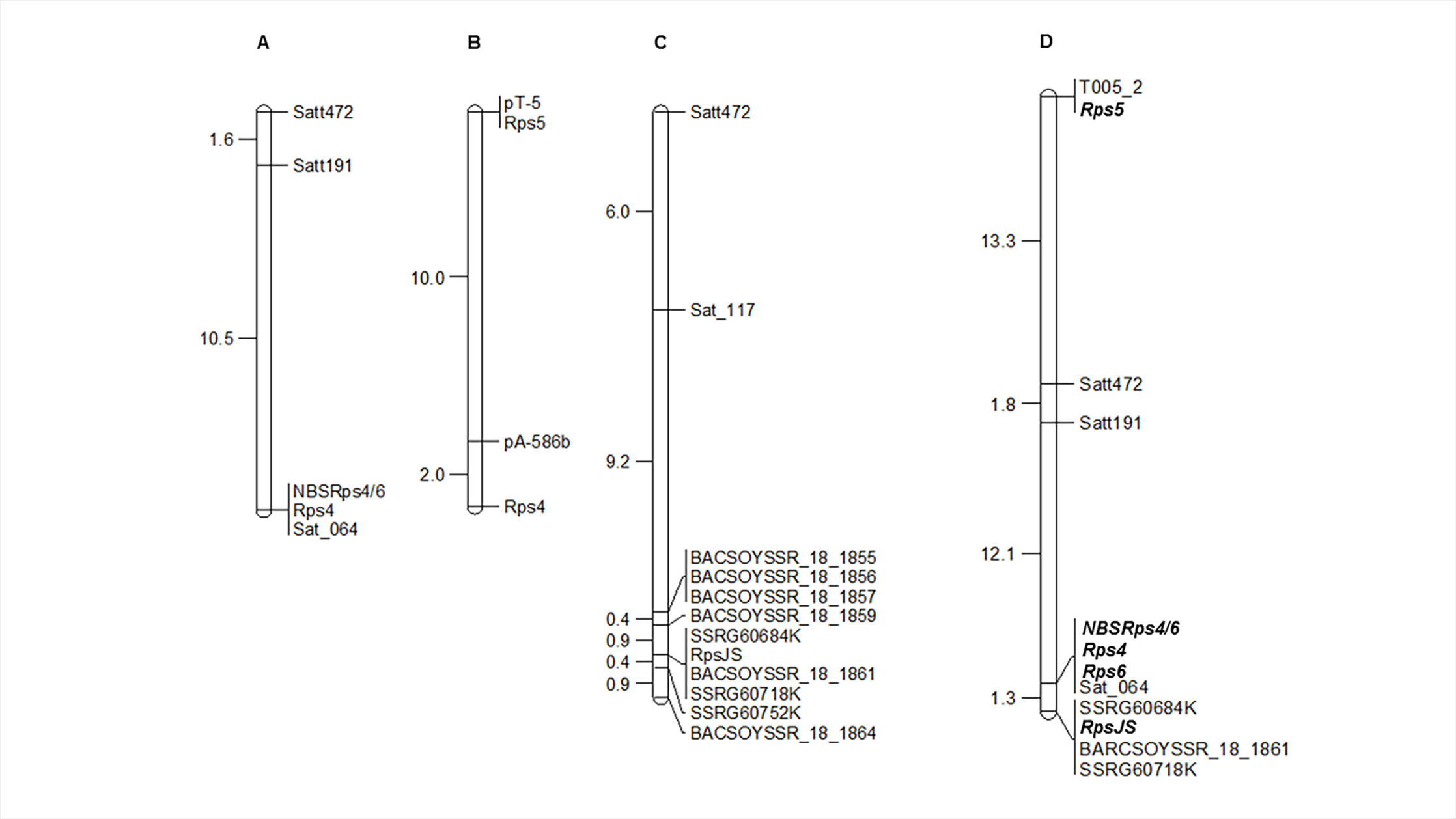

Supplement: S1 Fig — (A) The genetic map of the Rps4/6 region from the study by Sandhu et al. (2004) [50]. (B) The genetic map of the Rps4 and Rps5 region from Diers et al. (1992) [53]. (C) The genetic linkage map of the RpsJS region from the study of Sun et al. (2014) [54]. (D) The composite genetic map of the Rps loci located in the lower arm of Chromosome 18. The map was developed from three maps shown in A, B and C, and the co-segregation of Rps4 and Rps6 was from the study of Sandhu et al. (2004) [50]. (TIF) [file pone.0169950.s001.tif]
