# Supplementary material for: A Novel Phytophthora sojae Resistance Rps12 Gene Mapped to a Genomic Region That Contains Several Rps Genes
Source: PLoS One. 2017 Jan 12;12(1):e0169950. doi: 10.1371/journal.pone.0169950 (PMC5233422; doi:10.1371/journal.pone.0169950)
Supplement: S1 Table — (DOCX) [file pone.0169950.s002.docx]

**S1 Table. Twenty-seven *Rps* genes that confer resistance to *Phythopthora sojae* in soybean.**

| ***Rps* gene** | **Chromosome** | **Molecular linkage group** |
| --- | --- | --- |
| *Rps1a, b, c, d, k* | 3 | N [21, 24, 32, 45]* |
| *Rps2* | 16 | J [41] |
| *Rps3a, b, c* | 13 | F [45-46] |
| *Rps4* | 18 | G [51] |
| *Rps5* | 18 | G [52] |
| *Rps6* | 18 | G [49] |
| *Rps7* | 3 | N [4] |
| *Rps8* | 13 | F [42-43, 48] |
| *Rps9* | 3 | N [25] |
| *Rps10* | 17 | D2 [55] |
| *Rps11* | 7 | M [58] |
| *UN1* | 3 | N [40] |
| *UN2* | 16 | J [40] |
| *Yu25* | 3 | N [30] |
| *YD29* | 3 | N [26] |
| *YD25* | 3 | N 39] |
| *YB30* | 19 | L [57] |
| *RpsZS18* | 2 | D1b [56] |
| *RpsSN10* | 13 | F [44] |
| *Rps1?* | 3 | N [14] |
| *RpsJS* | 18 | G [54] |
|  |  |  |

*, References are shown in parentheses.
