# Supplementary material for: A Novel Phytophthora sojae Resistance Rps12 Gene Mapped to a Genomic Region That Contains Several Rps Genes
Source: PLoS One. 2017 Jan 12;12(1):e0169950. doi: 10.1371/journal.pone.0169950 (PMC5233422; doi:10.1371/journal.pone.0169950)
Supplement: S2 Table — (DOC) [file pone.0169950.s003.doc]

**S2 Table. Primers for microsatellite and NBSRps4/6-sequence-specific markers**

| **Sl. No.** | **Name of Primers** |  | **Sequence of primers (5’ to 3’)** | **Annealing temp. (°C)** | **Population AX20925** |
| --- | --- | --- | --- | --- | --- |
| 1 | Satt472 (BARCSOYSSR_18_1708) | F  R | GCGAATACATAAAACTCAAATTCAAATCATA  GCGTTCTATAAATTTCATTCATAGTTTCAAT | 55 | — |
| 2 | Satt191 (BARCSOYSSR_18_1750) | F  R | CGCGATCATGTCTCTG  GGGAGTTGGTGTTTTCTTGTG | 55 | + |
| 3 | BARCSOYSSR_18_1755 | F  R | TGCATTGTGTAAAACAAATTCAC  CATGCTCACACCAGAAGAGG | 47 | + |
| 4 | BARCSOYSSR_18_1760 | F  R | TCCCAAACATGTGAACCAAA  CGCACACGCGGAAATAATA | 48 | — |
| 5 | BARCSOYSSR_18_1765 | F  R | ACCGAAGTTCTCTGGAGTCG  CGGAGTACTTAAAACATAGTCAACC | 50 | + |
| 6 | Sat_117 (BARCSOYSSR_18_1767) | F  R | TTTGGCAGTTTCTTGTAG  GCTGGATCGCAGTTA | 47 | — |
| 7 | BARCSOYSSR_18_1770 | F  R | GGGCATAGATCCTTGTTGGA  TTAAGAGGTCAAGCCCCGTT | 50 | — |
| 8 | BARCSOYSSR_18_1790 | F  R | ATCTTGGGGGCAACTTTCAT  GCCTCTGAAAGAGAAAGGGAA | 50 | — |
| 9 | BARCSOYSSR_18_1795 | F  R | GGGTAAAAACAATTCTGCCTTT  TGTCTCCCTGCATAATCAATG | 48 | — |
| 10 | BARCSOYSSR_18_1800 | F  R | GGGGTTGTGCCCCTTATTTA  GAAGCGGAACTACGTTTTGG | 50 | — |
| 11 | BARCSOYSSR_18_1810 | F  R | CACGTGGGACGACTTCATAG  TGCTTTCAAAAACACACACACA | 50 | — |
| 12 | BARCSOYSSR_18_1820 | F  R | ACTTTGATTCCCATGCAAGC  CTTAACCTGCAGCTTTTGCC | 50 | + |
| 13 | BARCSOYSSR_18_1830 | F  R | TTTATCCTGGGGACTTCGTG  GGACATTTGGACTCTCCTCCT | 50 | + |
| 14 | BARCSOYSSR_18_1840 | F  R | ACCATTTTCCCCCTTAATGC  CCAGATTGTTCAATACCGCA | 48 | + |
| 15 | BARCSOYSSR_18_1850 | F  R | AAATGCATTCGTGGCTTTCT  TGGGTATTATTTGGCAAGCAC | 48 | — |
| 16 | Sct_187 (BARCSOYSSR_18_1853) | F  R | CATGCTCCCATTCTCT  AACATTGGCTTTTTACTTAG | 47 | — |
| 17 | BARCSOYSSR_18_1855 | F  R | CGTAAAACCATGGTCGTCAA  AATGCATGGGGTGTGAATTT | 50 | — |
| 18 | BARCSOYSSR_18_1856 | F  R | TGGCCATATGCCTAGCTGAT  ATGGTGAGCAAACGTCATTG | 50 | — |
| 19 | BARCSOYSSR_18_1857 | F  R | TTTTTCAATGAAGGTGGAATCT  CGGATGCGCCTTTTATTATT | 45 | — |
| 20 | NBSRps4/6-533 | F  R | GCACGAGGCTTTCGTTCTCTAGTCTG  GGGGATGCTCCCCAAAATTGACTCAGTC | 55 | + |
| 21 | NBSRps4/6-130 | F  R | GGGAGCATCCCAAGTTTGTAGTTATTC  GGGGATGCTCCCCAAAATTGACTCAGTC | 55 | + |
| 22 | NBSRps4/6-1272 | F  R | GCACGAGGCTTTCGTTCTCTAGTCTG  CCCCAACTCACTTCCTTGATTCTTGAC | 55 | — |
| 23 | NBSRps4/6-869 | F  R | GGGAGCATCCCAAGTTTGTAGTTATTC  CCCCAACTCACTTCCTTGATTCTTGAC | 55 | — |
| 24 | Sat_064 (BARCSOYSSR_18_1858) | F  R | TAGCTTTATAATGAGTGTGATAGAT  GTATGCAAGGGATTAATTAAG | 47 | + |
| 25 | BARCSOYSSR_18_1859 | F  R | CTCAATCGCATCCTTGCATA  GCCTTCCAACTCATGTTTCAA | 48 | + |
| 26 | BARCSOYSSR_18_1860 | F  R | AGACATTCGTTGCAAAAGCC  TTAGCCCTTCCCAAGAAACA | 50 | + |
| 27 | SSRG60684K | F  R | CAATGTTTTACTTTGGAGC  AAAATAAATACTAATGTAAAATGAT | 42 | + |
| 28 | BARCSOYSSR_18_1861 | F  R | TGCCACAATGTCCACAACTT  CCCTTTTCTTTTGCCTCTCC | 50 | + |
| 29 | SSRG60718K | F  R | TTTCTTAGCCTTGTACTTTC  CCAAAGGCTGTATCTGTT | 42 | — |
| 30 | SSRG60752K | F  R | AACAACACCATCAACAAAACGAAA  GTTCACGGACGAGGAAGATA | 50 | — |
| 31 | BARCSOYSSR_18_1870 | F  R | TCTCTTCCTCACCCCATGAT  GCAGCTTCTTGGTCTCAAGC | 50 | — |
| 32 | Sat_372 | F  R | GCGTCTCGAGGTAATTATCTATTTATCTTTT  GCGAGTTTGGTAACATCGAGTATTGAT | 50 | — |
| 33 | BARCSOYSSR_18_1880 | F | AAATGGAAACACACCGATTG | 48 | — |
|  |  | R | GCTGTTACCTCAGAGATTCTTTCT |  |  |
| 34 | BARCSOYSSR_18_1890 | F  R | TGTTAGTGTACGCGTTACAAAATATAA  AAAGTGCATGTACATTAGTGAATTTTA | 48 | — |
| 35 | BARCSOYSSR_18_1900 | F  R | TGCCATTGTGGTTCTTCTTTT  TTTTCTTTTTAAAGGTGTGCCA | 48 | + |
| 36 | BARCSOYSSR_18_1910 | F  R | TCATTCAAACATTAATAGTCAACAAA  TGGTTAGTTCTAGGAATTTGGTCTC | 47 | + |
| 37 | BARCSOYSSR_18_1930 | F  R | TTACAGCATTTATCTCCGTATTGA  ACATTTTCTAAAATTAAATTCCTACCA | 45 | + |
| 38 | BARCSOYSSR_18_1950 | F  R | ACATTTTGGCGCATTTTAGC  CCTTTAGAAGCCGATTTCCC | 48 | — |

+, Polymorphic.

- , Non polymorphic.

Sequences of primers for SSR markers are based on SOYBASE (http://www. Soybase.org/). Sequences of NBSRps4/6 primers are based on the NBSRps4/6 sequence that mapped to *Rps4* [50].
