# Supplementary material for: A Novel Phytophthora sojae Resistance Rps12 Gene Mapped to a Genomic Region That Contains Several Rps Genes
Source: PLoS One. 2017 Jan 12;12(1):e0169950. doi: 10.1371/journal.pone.0169950 (PMC5233422; doi:10.1371/journal.pone.0169950)
Supplement: S3 Table — (DOC) [file pone.0169950.s004.doc]

**S3 Table.** ^a^Phenotypes and ^b^genotypes of 120 AX20925 RILs.

|  | **Response to**  ***P. sojae* isolates** | | **Molecular markers** | | | | | | | | | | | | |
| --- | --- | --- | --- | --- | --- | --- | --- | --- | --- | --- | --- | --- | --- | --- | --- |
| **RILs** | **R17+**  **Val**  **12-11** | **P7074** | **Satt191** | **18_1755** | **18_1765** | **18_1820** | **18_1830** | **18_1840** | **NBSLRR**  **130** | **NBSLRR**  **533** | **Sat_064** | **18_1859** | **18_1860** | **SSRG**  **60684K** | **18_1861** |
| PI399036 | B | B | B | B | B | B | B | B | B | B | B | B | B | B | B |
| AR2 | A | A | A | A | A | A | A | A | A | A | A | A | A | A | A |
| 1 | B | B | B | B | B | B | B | B | B | B | B | B | B | B | B |
| 2 | A | A | A | A | A | A | A | A | A | A | A | A | A | A | A |
| 3 | B | B | A | A | A | B | B | B | B | B | B | B | B | B | B |
| 4 | B | B | B | B | B | B | B | B | B | B | B | B | B | B | B |
| 5 | B | B | B | B | B | B | B | B | B | B | B | B | B | B | B |
| 6 | B | B | A | A | A | A | B | B | A | A | A | A | A | A | A |
| 7 | B | B | B | B | B | B | B | B | B | B | B | B | B | B | B |
| 8 | B | B | B | B | B | B | B | B | B | B | B | B | B | B | B |
| 9 | B | B | B | B | B | B | B | B | A | A | A | A | A | A | A |
| 10 | B | B | A | A | A | A | A | A | B | B | B | B | B | B | B |
| 11 | B | B | B | B | B | B | B | B | B | B | B | B | B | B | B |
| 12 | B | B | B | B | B | B | B | B | B | B | B | A | A | A | A |
| 13 | B | B | B | B | B | B | B | B | B | B | B | B | B | B | B |
| 14 | B | B | B | B | B | B | B | B | B | B | B | B | B | B | B |
| 15 | B | B | B | B | B | B | B | B | B | B | B | B | B | B | B |
| 16 | B | B | A | A | A | A | A | A | A | A | A | A | A | A | A |
| 17 | B | B | B | B | B | B | B | B | B | B | B | B | B | B | B |
| 18 | B | B | A | A | A | A | A | A | B | B | B | B | B | B | A |
| 19 | B | B | B | B | B | B | B | B | B | B | B | B | B | B | B |
| 20 | B | B | B | B | B | B | B | B | B | B | B | B | B | B | B |
| 21 | B | B | B | B | B | B | B | B | B | B | B | B | B | B | B |
| 22 | B | B | A | A | A | A | A | B | B | B | B | B | B | B | B |
| 23 | B | B | B | B | B | B | B | B | B | B | B | B | B | B | B |
| 24 | A | A | A | A | A | A | A | A | A | A | A | A | A | A | A |
| 25 | B | B | B | B | B | B | B | B | B | B | B | B | B | B | B |
| 26 | A | A | B | B | B | B | B | B | A | A | A | A | A | A | A |
| 27 | B | B | A | A | A | A | B | B | B | B | B | B | B | B | B |
| 28 | B | B | A | A | A | A | A | A | B | B | B | B | B | B | B |
| 29 | B | B | B | B | B | B | B | B | B | B | B | B | B | B | B |
| 30 | B | B | B | B | B | B | B | B | B | B | B | B | B | B | B |
| 31 | B | B | B | B | B | B | B | B | B | B | B | B | B | B | B |
| 32 | B | B | B | B | B | B | B | B | B | B | B | B | B | B | B |
| 33 | B | B | A | A | A | A | B | B | B | B | B | B | B | B | B |
| 34 | B | B | B | B | B | B | B | B | B | B | B | B | B | B | B |
| 35 | B | B | A | A | A | B | B | B | B | B | B | B | B | B | B |
| 36 | A | A | A | A | A | A | A | A | A | A | A | A | A | A | A |
| 37 | B | B | B | B | B | B | B | B | B | B | B | B | B | B | B |
| 38 | A | A | A | A | A | A | A | A | A | A | A | A | A | A | A |
| 39 | B | B | B | B | B | B | B | B | B | B | B | B | B | B | B |
| 40 | B | B | B | B | B | B | B | B | B | B | B | B | B | B | B |
| 41 | A | A | A | A | A | A | A | A | A | A | A | A | A | A | A |
| 42 | B | B | A | A | A | A | B | B | B | B | B | A | A | A | A |
| 43 | B | B | B | B | B | B | B | B | B | B | B | B | B | B | B |
| 44 | A | A | A | A | A | A | A | A | A | A | A | A | A | A | A |
| 45 | B | B | A | A | A | A | B | B | B | B | B | A | A | A | A |
| 46 | B | B | B | B | B | B | B | B | B | B | B | B | B | B | B |
| 47 | B | B | B | B | B | B | B | B | B | B | B | B | B | B | B |
| 48 | B | B | B | B | B | B | B | B | B | B | B | B | B | B | B |
| 49 | B | B | B | B | B | B | B | B | B | B | B | A | A | B | B |
| 50 | B | B | B | B | B | B | B | B | B | B | B | B | B | B | B |
| 51 | B | B | B | B | B | B | B | B | B | B | B | B | B | B | B |
| 52 | B | B | B | B | B | B | B | B | B | B | B | B | B | B | B |
| 53 | B | B | A | A | A | A | B | B | B | B | B | B | B | B | B |
| 54 | B | B | B | B | B | B | B | B | B | B | B | B | B | B | B |
| 55 | B | B | B | B | B | B | B | B | B | B | B | B | B | B | B |
| 56 | B | B | A | A | A | B | B | B | B | B | B | B | B | B | B |
| 57 | A | A | A | A | A | A | A | A | A | A | A | A | A | A | A |
| 58 | B | B | B | B | B | B | B | B | B | B | B | B | B | B | B |
| 59 | B | B | B | B | B | B | B | B | B | B | B | B | B | B | B |
| 60 | B | B | B | B | B | B | B | B | B | B | B | B | B | B | B |
| 61 | A | A | A | A | A | A | A | A | A | A | A | A | A | A | A |
| 62 | A | A | A | A | A | A | A | A | A | A | A | A | A | A | A |
| 63 | A | A | A | A | A | A | A | A | A | A | A | A | A | A | A |
| 64 | A | A | A | A | A | A | A | A | A | A | A | A | A | A | A |
| 65 | A | A | A | A | A | A | A | A | A | A | A | A | A | A | A |
| 66 | A | A | A | A | A | A | B | B | A | A | A | A | A | A | A |
| 67 | B | B | B | B | B | B | B | B | B | B | B | B | B | B | B |
| 68 | A | A | B | B | B | A | A | A | A | A | A | A | A | A | A |
| 69 | A | A | A | A | A | A | A | A | A | A | A | A | A | A | A |
| 70 | A | A | A | A | A | A | B | B | A | A | A | A | A | A | A |
| 71 | A | A | A | A | A | A | A | A | A | A | A | A | A | A | A |
| 72 | A | A | A | A | A | A | A | A | A | A | A | A | A | A | A |
| 73 | A | A | A | A | A | A | A | A | A | A | A | A | A | A | A |
| 74 | A | A | A | A | A | A | A | A | A | A | A | A | A | A | A |
| 75 | A | A | A | A | A | A | A | A | A | A | A | A | A | A | A |
| 76 | A | A | A | A | A | A | A | A | A | A | A | A | A | A | A |
| 77 | A | A | A | A | A | A | A | A | A | A | A | A | A | A | A |
| 78 | A | A | A | A | A | A | A | A | A | A | A | A | A | A | A |
| 79 | A | A | A | A | A | A | A | A | A | A | A | A | A | A | A |
| 80 | A | A | A | A | A | A | A | A | A | A | A | A | A | A | A |
| 81 | A | A | A | A | A | A | A | A | B | B | B | B | B | B | B |
| 82 | B | B | B | B | B | B | B | B | B | B | B | B | B | B | B |
| 83 | A | A | B | B | A | A | A | A | A | A | A | A | A | A | A |
| 84 | A | A | A | A | A | A | A | A | A | A | A | A | A | A | A |
| 85 | A | A | A | A | A | A | A | A | A | A | A | A | A | A | A |
| 86 | A | A | A | A | A | A | A | A | B | B | A | A | A | A | A |
| 87 | B | B | B | B | B | B | B | B | B | B | B | B | B | B | B |
| 88 | A | A | A | A | A | A | A | A | A | A | A | A | A | A | A |
| 89 | A | A | B | B | B | A | A | A | A | A | A | A | A | A | A |
| 90 | A | A | A | A | A | A | A | A | A | A | A | A | A | A | A |
| 91 | A | A | B | B | B | A | A | A | A | A | A | A | A | A | A |
| 92 | A | A | A | A | A | A | A | A | A | A | A | A | A | A | A |
| 93 | A | A | A | A | A | A | A | A | A | A | A | A | A | A | A |
| 94 | A | A | A | A | A | A | A | A | A | A | A | A | A | A | A |
| 95 | A | A | A | A | A | A | A | A | A | A | A | A | A | A | A |
| 96 | A | A | B | B | B | A | A | A | A | A | A | A | A | A | A |
| 97 | A | A | A | A | A | A | A | A | A | A | A | A | A | A | A |
| 98 | A | A | A | A | A | A | A | A | A | A | A | A | A | A | A |
| 99 | B | B | B | B | B | B | B | B | B | B | B | B | B | B | B |
| 100 | A | A | B | B | B | B | B | A | A | A | A | A | A | A | A |
| 101 | B | B | B | B | B | B | B | B | B | B | B | B | B | B | B |
| 102 | A | A | B | B | B | A | A | A | A | A | A | A | A | A | A |
| 103 | A | A | A | A | A | A | A | A | A | A | A | A | A | A | A |
| 104 | A | A | A | A | A | A | A | A | A | A | A | A | A | A | A |
| 105 | A | A | A | A | A | A | A | A | A | A | A | A | A | A | A |
| 106 | A | A | A | A | A | A | A | A | A | A | A | A | A | A | A |
| 107 | A | A | A | A | A | A | A | A | A | A | A | A | A | A | A |
| 108 | A | A | A | A | A | A | A | A | A | A | A | A | A | A | A |
| 109 | A | A | A | A | A | A | A | A | A | A | A | A | A | A | A |
| 110 | A | A | A | A | A | A | A | A | A | A | A | A | A | A | A |
| 111 | A | A | A | A | A | A | A | A | A | A | A | A | A | A | A |
| 112 | B | B | B | B | B | B | B | B | B | B | B | B | B | B | B |
| 113 | A | A | A | A | A | A | A | A | A | A | A | A | A | A | A |
| 114 | A | A | A | A | A | A | A | A | A | A | A | A | A | A | A |
| 115 | A | A | A | A | A | A | A | A | A | A | A | A | A | A | A |
| 116 | A | A | B | B | B | A | A | A | A | A | A | A | A | A | A |
| 117 | A | A | A | A | A | A | A | A | A | A | A | A | A | A | A |
| 118 | A | A | A | A | A | A | A | A | A | A | A | A | A | A | A |
| 119 | A | A | B | A | A | A | A | A | A | A | A | A | A | A | A |
| 120 | B | B | B | B | B | B | B | B | B | B | B | B | B | B | B |

^a^Responses of individual 120 RILs to (i) R17 + Val 12-11, the mixture of *P. sojae* R17 and Val 12-11 isolates; (ii) *P. sojae* P7074 isolate. A, susceptible response; B, resistant response.

^b^Genotypes at the SSR marker sites: A, homozygous for AR2 allele; B, homozygous for PI399036 allele.
